# Submassive Pulmonary Embolism: A Re-evaluation of Hemodynamic Instability

**DOI:** 10.7759/cureus.4644

**Published:** 2019-05-11

**Authors:** Megan Obi, Clifford D Packer

**Affiliations:** 1 Internal Medicine, Case Western Reserve University School of Medicine, Cleveland, USA

**Keywords:** pulmonary embolism, pe, thrombolytics, hemodynamic instability, anticoagulation

## Abstract

Current medical management of pulmonary embolism (PE) is driven by risk stratification, with thrombolytic treatment reserved for patients with hemodynamic instability. We describe a case of a man with acute submassive bilateral pulmonary emboli and a right popliteal deep vein thrombosis (DVT), who had persistent shortness of breath, tachycardia, and hypoxemia but remained normotensive and was therefore not treated with thrombolytics until he suffered a fatal cardiac arrest on hospital day six. We examine the indications, risks, and potential benefits of thrombolytic treatment in patients with submassive PE who exhibit signs of instability but do not meet current indications for thrombolytic treatment with persistent hypotension or shock.

## Introduction

The incidence of pulmonary embolism (PE) ranges from 70 to 300 cases per 100,000 persons per year, with mortality rates of approximately 40% in cases of high risk and hemodynamically unstable patients. Current guidelines recommend the use of thrombolytic treatment only for patients with hemodynamic instability defined as persistent hypotension or shock (systolic blood pressure <90 mmHg or a decrease in diastolic blood pressure ≥40 mmHg) [1-2]. However, a subset of patients remain normotensive while exhibiting other signs and symptoms of instability and deterioration, such as persistent chest pain, tachycardia, and hypoxemia. We consider the evidence as to whether these “semi-stable” PE patients might also benefit from thrombolytic treatment.

## Case presentation

**Figure 1:**
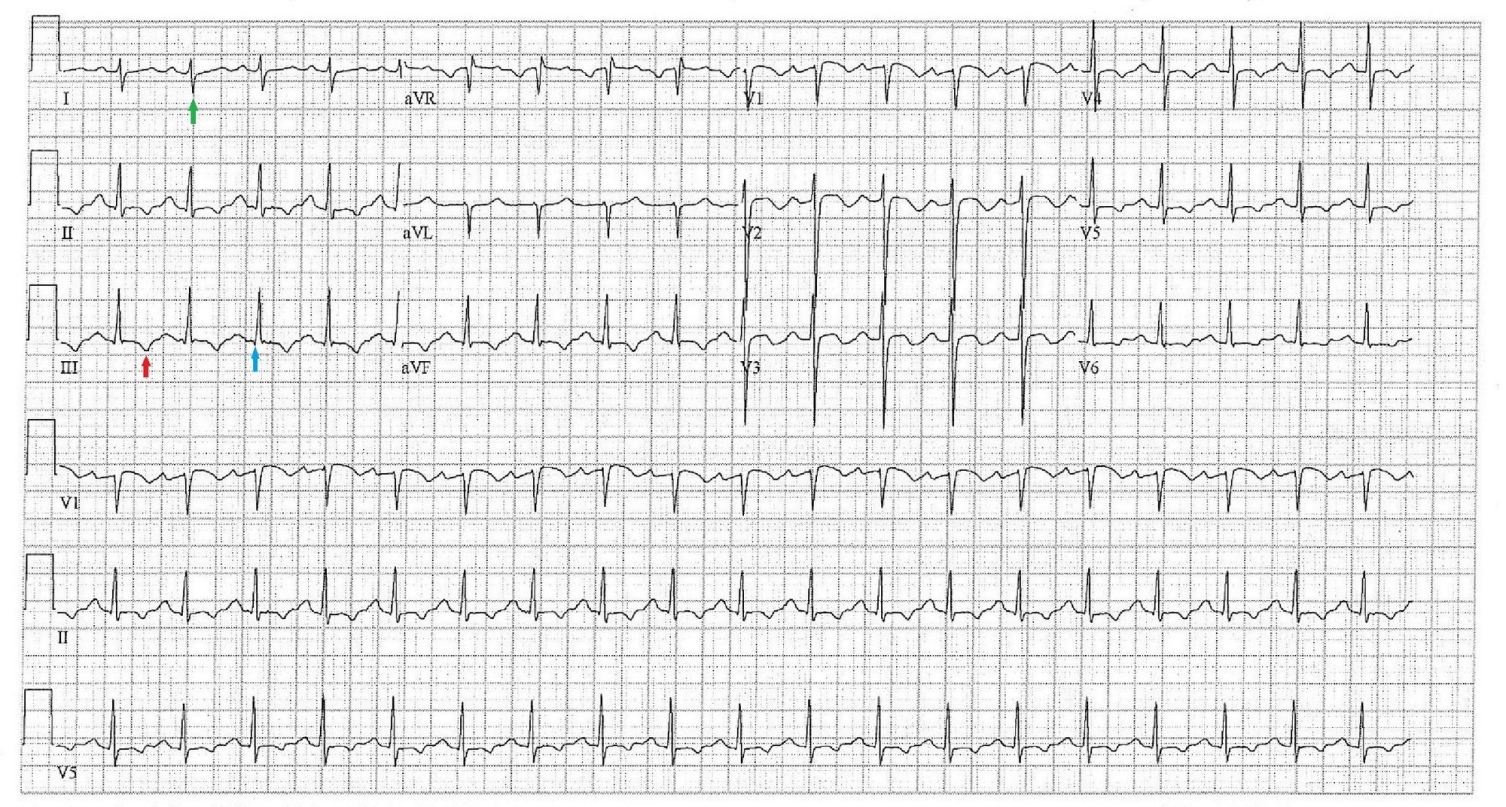
Electrocardiogram demonstrating deep S wave in lead I (green arrow), small Q wave (blue arrow) and inverted T wave (red arrow) in lead III

A 55-year-old man with a history of type II diabetes mellitus, hypertension, obstructive sleep apnea, and depression developed pleuritic chest pain and shortness of breath, and had a syncopal episode during a car trip from Texas to Cleveland. In the emergency room (ER), his blood pressure was 120/83 mmHg, pulse 119/min, respiratory rate 22/min, temperature 36.6^o^C, and oxygen (O_2_) saturation was 93% on room air. Physical exam was notable for an obese male in no acute distress with tachycardia, regular rhythm, clear lungs, shallow breaths without accessory muscle usage, and mild epigastric tenderness. His electrocardiogram (ECG) demonstrated sinus tachycardia at 117 bpm with a deep S wave in lead I, a small Q wave and inverted T wave in lead III (Figure 1).

**Figure 2:**
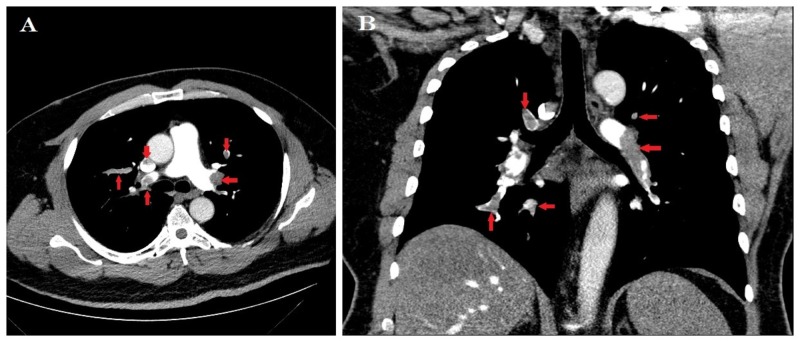
Axial (A) and sagittal (B) contrast-enhanced computed tomography pulmonary angiogram images demonstrating multiple bilateral filling defects (arrows) suggestive of pulmonary emboli

Initial laboratory testing was significant for serum sodium 132 mmol/L, creatinine 1.6 mg/dL, ProBNP 3188pg/mL, and initial troponin 0.12, peaking at 0.2 on hospital day two. Computed tomography (CT) chest showed bilateral submassive pulmonary emboli in multiple branches of the right and left upper and lower lobe pulmonary arteries, with no indication of a saddle embolus or main pulmonary artery thrombus (Figures 2A-2B).

The patient was started on an intravenous (IV) heparin drip and thrombolytics were deferred due to perceived hemodynamic stability and timing of presentation. While in the ER, he became more tachycardic with heart rate 130-140 bpm, and developed jugular venous distention and acute hypoxemia requiring 3 liters (L) nasal cannula to maintain normal O_2_ saturation. He was admitted to the medical intensive care unit (MICU) due to possible signs of acute decompensation.

In the MICU, ultrasound revealed a right popliteal deep vein thrombosis (DVT); an inferior vena cava filter was considered, but the patient was considered hemodynamically stable and placement of a filter was deferred. Over the second and third hospital days, he remained tachycardic at 105-130/min with blood pressures in the 110/80 mmHg range and stable O_2_ saturations on 3L per nasal cannula. Transthoracic echocardiogram revealed right ventricular strain and a mobile structure in the right atrium, possibly consistent with thrombus. On hospital day four, he remained hemodynamically stable but experienced mild pleuritic chest pain and shortness of breath. On hospital day five, the patient was switched from IV heparin to enoxaparin 120 mg twice daily with a two-hour overlap; he was noted to have a transient O_2_ desaturation but remained normotensive. In the early hours of day six, he became diaphoretic and developed sustained tachycardia at 140/min with a blood pressure of 100/70 mmHg. He then developed chest pain and worsening dyspnea and lethargy, and was found to be in atrial flutter with 2:1 block. In the early afternoon, while speaking with the medical team and later while working with physical therapy, the patient complained of worsening shortness of breath, and was noted to be diaphoretic and persistently tachycardic at 120/min. Shortly thereafter, he lost consciousness and was found to be in cardiac arrest. He was treated per advanced cardiac life support (ACLS) protocol with temporary restoration of circulation, but became asystolic and was pronounced dead after 30 minutes.

## Discussion

PE are risk stratified into three categories: high-risk (or “massive”) defined by hemodynamic instability with sustained systolic blood pressure (SBP) <90 mmHg or a decrease in SBP by ≥ 40 mmHg from baseline, intermediate risk (or “submassive”) defined by hemodynamic stability with SBP ≥90 mmHg and features of right heart dysfunction or myocardial necrosis, and low risk [1]. Approximately 20%-25% of patients with PEs present with submassive emboli and mortality rates for intermediate risk PE range from 3% to 15% if imaging or biomarker indications of right heart dysfunction are present [3]. Our patient was categorized as having bilateral submassive PE with right heart strain, possible right atrial thrombus, and a subsequent type II myocardial infarction. While in the hospital, he was never officially considered “high-risk” due to persistent normotension. However, he did have persistent tachycardia and hypoxemia, atrial flutter, progressive fatigue, dyspnea on minimal exertion, persistent pleuritic chest pain, and diaphoresis. Despite these worrisome signs and symptoms (which persisted and worsened over his six-day course), he was classified as intermediate risk and treated only with IV heparin with transition to enoxaparin anticoagulation on hospital day five.

Given the significant mortality associated with PEs, a number of studies have retrospectively assessed various risk factors including advanced age, prolonged immobility, malignancy, polytrauma, recent major surgery, heart failure, prior venous thromboembolism, and dyslipidemia that can be utilized as diagnostic indicators [4]. As for clinical risk stratification tools, the pulmonary embolism severity index (PESI) and its simplified version, sPESI, include assessment of age, sex, health history, vitals, and presence of alteration in mental status as factors to estimate 30-day mortality risk. Our patient’s sPESI score based on tachycardia alone, as well as his PESI score based on his age, sex, heart rate, mental and pulmonary status, classified him as high-risk and thus at significantly increased risk of 30-day mortality. While this tool does indicate risk for potential decompensation and mortality, its current primary role has been in identifying low-risk patients who might be eligible for outpatient treatment. For high-risk patients, close monitoring is recommended, but specific management recommendations are not defined [5-6].

Systemic thrombolysis is considered standard aggressive therapy for high-risk PE. However, for intermediate-risk patients, thrombolytic use is considered controversial, and conservative therapy with anticoagulation is most commonly utilized. Early research initially found that utilization of thrombolytics had a favorable effect in the treatment of hemodynamically stable major PE and thus subsequent recent studies have analyzed the risks and benefits of thrombolytic treatment in intermediate risk patients [7]. The Pulmonary Embolism International Thrombolysis (PEITHO) trial found that up-front thrombolytic therapy decreased the risk of hemodynamic decompensation by 3.4% in patients with submassive PEs with the additional risk markers of right ventricle (RV) dysfunction and elevated troponins. However, the risks of extracranial (5.1%) and intracranial (1.8%) bleeding complications exceeded the benefit for these patients [8]. The Ultrasound Accelerated Thrombolysis of Pulmonary Embolism (ULTIMA) trial found that use of standardized low dose ultrasound assisted catheter-directed thrombolysis (USAT) resulted in hemodynamic and echocardiographic risk index improvement as compared to heparin anticoagulation alone in patients with acute PE with RV dysfunction. Other than a couple of instances of transient hemoptysis and an access-site hematoma, no major bleeding episodes occurred with the use of USAT [9-10]. The American Heart Association guidelines suggest consideration of fibrinolysis in patients with submassive PE with adverse prognosis, but randomized trial data to guide practice have been limited, and the clinical definition of “adverse prognosis” has not yet been clearly defined in the literature [1]. There are few if any reports of what clinical deterioration looks like in a hemodynamically stable patient that may be indicative of increased risk of mortality from clot burden and thus warrant thrombolytic consideration [2]. While the American College of Cardiology notes that common clinical sequelae of submassive PE include rhythm disturbances such as right bundle branch block and atrial arrhythmias, signs of pulmonary hypertension such as chronic dyspnea, syncope, and chest discomfort, and signs of RV failure such as peripheral edema, ascites, and nocturia (many which were seen in our patient), the prognostic significance of these findings remains unclear [11].

Cho et al. argue that criteria for RV dysfunction and hemodynamic instability may be considered too stringent in some cases when assessing short-term mortality and as such, some patients deemed hemodynamically stable due to their normal blood pressure may actually be high risk for clinical deterioration and subsequent instability, as was clearly the case in our patient [12]. Stewart et al. found that in the context of submassive PE, fibrinolysis has the greatest potential for preventing deterioration in patients most likely to have significantly impaired quality of life in part due to their comorbidities [13]. In the context of our patient, these comorbid conditions have been found to include obesity or coexisting cardiopulmonary conditions, indicating a potential benefit of thrombolytic treatment for our patient.

Choi et al. contend that risk tools, notably the PESI, should be more widely used in conjunction with assessment of hemodynamic stability and echocardiographic findings in order to identify and target high-risk patients [14]. In the context of our case, it is unclear whether the clinical data assessed in the PESI is sufficient to identify high-risk patients who would clearly benefit from thrombolytic treatment. More research is needed to better understand how the various clinical sequelae of PE (i.e., arrhythmias, progressive dyspnea, chest pain, hypoxemia, and evidence of RV dysfunction or strain) might predict acute decompensation over time. While the risk of thrombolytics, the invasive nature of embolectomy, and the uncertainty of benefit of inferior vena cava filters remain troublesome constants, novel research is underway to assess the efficacy and safety of methods such as ultrasound-assisted vs. standard catheter-directed delivery of thrombolysis, as well as novel oral anticoagulants as alternatives to systemic fibrinolysis and standard anticoagulation [9,15-17]. This case serves as an indication that we may need to reconsider and perhaps redefine our PE risk categories, taking into account not only the presence of high-risk symptoms, but the persistence of these symptoms over time without improvement. 

## Conclusions

Treating submassive pulmonary emboli can be very challenging. There may be subgroups of patients with submassive PE without hemodynamic instability (defined as hypotension and shock) that would benefit from thrombolytic treatment. Patients such as ours, with multiple clinical indicators of clinical instability that persist or worsen over several days despite appropriate anticoagulation, might be a particularly high-risk subgroup. Pending further clinical research, physicians should not only consider current guidelines but also take into consideration the clinical manifestations of instability over time in making treatment decisions for patients with submassive PE.
